# Endothelial-to-Mesenchymal Transition (EndoMT): Roles in Tumorigenesis, Metastatic Extravasation and Therapy Resistance

**DOI:** 10.1155/2019/8361945

**Published:** 2019-08-01

**Authors:** Valentin Platel, Sébastien Faure, Isabelle Corre, Nicolas Clere

**Affiliations:** ^1^Micro & Nanomédecines Translationnelles-MINT, Univ Angers, INSERM U1066, CNRS UMR 6021, Angers, France; ^2^Sarcomes Osseux et Remodelage des Tissus Calcifiés Phy-OS, Université de Nantes INSERM UMR U1238, Faculté de Médecine, F-44035 Nantes, France

## Abstract

Cancer cells evolve in a very complex tumor microenvironment, composed of several cell types, among which the endothelial cells are the major actors of the tumor angiogenesis. Today, these cells are also characterized for their plasticity, as endothelial cells have demonstrated their potential to modify their phenotype to differentiate into mesenchymal cells through the endothelial-to-mesenchymal transition (EndoMT). This cellular plasticity is mediated by various *stimuli* including transforming growth factor-*β* (TGF-*β*) and is modulated dependently of experimental conditions. Recently, emerging evidences have shown that EndoMT is involved in the development and dissemination of cancer and also in cancer cell to escape from therapeutic treatment. In this review, we summarize current updates on EndoMT and its main induction pathways. In addition, we discuss the role of EndoMT in tumorigenesis, metastasis, and its potential implication in cancer therapy resistance.

## 1. Introduction

Since 2000, with the publication of *The Hallmarks of Cancer* by Hanahan and Weinberg [1], actualized in 2011 [2], considerable advancements have been done in the understanding of the biology of cancer. Importantly, a great effort has been made in the characterization of the microenvironment where evolve tumor cells. This microenvironment is composed of numerous cell types: immune cells (bone marrow-derived inflammatory cells, monocytes/macrophages, and lymphocytes), vascular cells (endothelial cells and pericytes), and stromal fibroblastic cells, and of an extracellular matrix composed of collagen and proteoglycans [3, 4]. While the importance of the stromal microenvironment in tumorigenesis has been recognized several decades ago [5], all the properties of the mobilized cells have not been described so far [6]. Among the cells identified in the tumor microenvironment, endothelial cells (ECs) are at the crossroad of different pathophysiological processes involved in tumor growth. Thus, since the initial studies conducted by Folkman [7, 8], numerous works have confirmed and studied the implication of ECs in the process of angiogenesis that is essential for optimal tumor progression [9, 10]. Besides their role in angiogenesis involving proliferation, migration, and adhesion, a new concept of endothelial plasticity has emerged last decade, as ECs have been described as able to modify their phenotype toward a mesenchymal profile. Initially characterized in physiological cardiac development, this plasticity is now described in not only several pathophysiological processes such as cardiac fibrosis [11], atherosclerosis [12], pulmonary hypertension [13], and vascular calcification but also in cancer [14, 15]. Besides this endothelial compartment, cancer-associated fibroblasts (CAFs) are the most abundant stromal cells in the tumor microenvironment and are critically involved in tumor progression. They actively interact with neoplastic cells and form a myofibroblastic microenvironment that promotes cancer growth and survival and supports malignancy. Thus, CAFs affect both the architecture and growth properties of the developing tumor. CAFs participate in the remodeling of peritumoral stroma, which is a prerequisite of neoplastic cell invasion, expansion, and metastasis. CAFs may originate from different sources (mesenchymal stromal cells (MSC), normal fibroblasts, and epithelial cells [16]) and also from ECs [17] through the process of endothelial-to-mesenchymal transition (EndoMT).

## 2. Main Features of Endothelial-to-Mesenchymal Transition (EndoMT)

EndoMT is defined as a cellular transition from an endothelial to a mesenchymal phenotype, owing from the plasticity potential of ECs. EndoMT was initially observed in 1975 in the formation of heart valves during embryogenesis in vertebrates from a detailed analysis of endocardial cytodifferentiation by transmission electron microscopy (TEM). In this last study, the authors followed the cardiac development of rat embryos and observed that, at E9.5, part of the endocardial cells from the atrioventricular canal and the efferent tract has a particular phenotype with morphological alterations such as cellular hypertrophy, lateralization of the Golgi apparatus, formation of cellular appendages, and loss of cell polarity [18]. These previous observations were subsequently confirmed in a chicken embryo model where the phenotypic change of cardiac ECs was correlated with new migratory properties and a concomitant expression of *α*-smooth actin (*α*-SMA) [19].

EndoMT is a transition process where ECs lose their endothelial characteristic features and acquire mesenchymal properties [20, 21]. In a similar way as in epithelial-mesenchymal transition (EMT), EndoMT is associated with the gain of mesenchymal markers such as N-cadherin, fibroblast-specific protein-1 (FSP-1), *α*-SMA, and types I/III collagen and with the corresponding loss of endothelial markers such as CD31 or platelet endothelial cell adhesion molecule 1 (PECAM1), Tie-2, and vascular endothelial (VE) cadherin [22] (Table 1).

**Figure 1:**
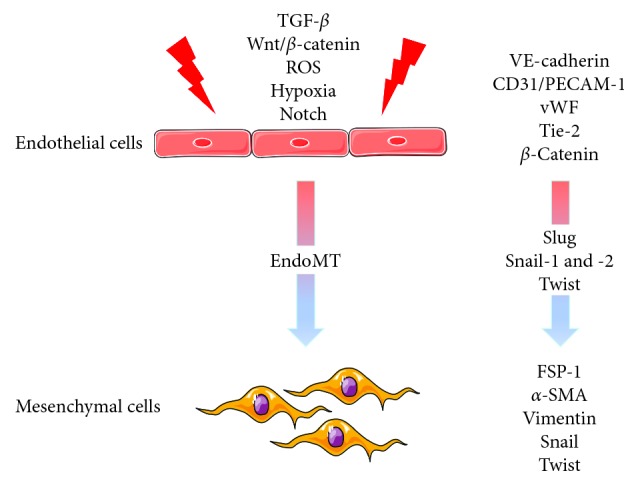
Phenotypic modifications during EndoMT. TGF-*β* (transforming growth factor-*β*); ROS (reactive oxygen species); VE cadherin (vascular endothelial cadherin); vWF (vonWillebrand factor); FSP-1 (fibroblast-specific protein-1); *α*-SMA (*α*-smooth muscle actin).

Aside from the acquisition of an activated profibrogenic phenotype, ECs further lose their apicobasal polarity and mesenchymal cells acquire new migratory properties. Furthermore, cells in transition have a proinflammatory secretory phenotype with increased secretion of cytokines such as IL-4, -13, -6, -8, and TNF-*α*, correlated with a synthesis of extracellular matrix proteins such as fibronectin and collagens [32]. Mechanistically, EndoMT is thought to be instigated by not only inductive signals like TGF-*β* [33], Wnt/*β*-catenin [27], and Notch [34] but also by hypoxia [13] and oxidative stress [35] (Figure 1).

EndoMT has been characterized in different endothelial human models such as tumor ECs isolated from prostate cancer [36], normal dermal microvascular endothelial cells (HDMECs), immortalized dermal microvascular endothelial HMEC-1 cells [37], human umbilical venous endothelial cells (HUVECs) [38], or human esophageal microvascular endothelial cells (HEMEC) [39] treated by TGF-*β*1 or *β*-2 or simultaneously by IL-1*β* and TGF-*β*2. Moreover, it has been reported that EndoMT can be induced by epigenetic modifications. Recently, in HUVECs, it has been demonstrated that combined knockdown of two ETS family transcription factors, *ERG* and *FLI*1, induces EndoMT coupled with dynamic epigenetic changes in ECs [40]. In these conditions, it has been demonstrated that (i) ECs are unable to form capillaries in Matrigel® and that (ii) cells not only lose their endothelial markers and acquire a mesenchymal phenotype but also become more invasive with increased migratory abilities [39]. In addition, analysis of cell proliferation reveals that ECs involved in EndoMT are able to progress through the cell cycle and that the acquisition of mesenchymal markers such as *α*-SMA is independent of cell cycling [38].

As described above, EndoMT has been confirmed through various studies conducted on different models of cultured endothelial cells treated by TGF-*β*1 or -*β*2. Some *in vivo* studies confirmed this process in not only various physiological situations (development [18, 41] and wound healing [42]) but also in pathological processes such as fibrotic diseases [43, 44], pulmonary hypertension [13], fibrodysplasia ossificans progressive disease [45], and in particular in cancer [46, 47]. The fact that EndoMT is a progressive, transitional, and complex process makes it difficult to explore *in vivo*, especially in fixed tissues. Nevertheless, EndoMT has been described in human samples of several pathologies. Detection of stromal cells coexpressing endothelial and mesenchymal markers was reported in patients with fibrotic disorders: cardiac fibrosis [48], radiation-induced rectal fibrosis [49], systemic sclerosis [50], and PHA [32]. Up to now, detection of EndoMT in human cancer patients has been reported in human colorectal tissue sections [51]. *In vivo* evidence of EndoMT has been rendered possible through the use of genetic lineage tracing technology, enabling to follow EC lineage conversion *in vivo* [52]. Use of Cre-LoxP-mediated endothelial tracing under the endothelial specific promoter (*Tie*-2 *or Cdh*5) has been used to develop animal models suitable for studying endothelial-to-mesenchymal transition. In non-cancerous pathologies, this strategy has been successful to show an EndoMT process in cardiac [53] and kidney [54, 55] fibrosis, in vein graft remodeling [56]. The endothelial origin of 40% of CAF has been demonstrated in a model of subcutaneous melanoma in *Tie*-2-*Cre* × R26TRosa Lox-stop-LacZ crossed mice [17]. Animal studies through endothelial-specific targeting revealed also the role of p53 in radiation-induced EndoMT in a pulmonary adenocarcinoma model [57]. In addition, in the murine pancreatic cancer model *Rip*-*Tag*2, absence of CD105/endoglin in ECs favored an EndoMT process [58].

## 3. EndoMT: A Transition Process with Heterogeneous Regulation

*In vitro* studies have provided a better understanding of phenotypic alterations of ECs during EndoMT and have also shown specificity of response depending on the experimental conditions. Thus, the expression of the mesenchymal proteins depends on (i) the nature of the inducing agent, (ii) the tissue origin of ECs [59], (iii) the signaling pathway(s) mobilized [60], and (iv) the cytokinic composition of the microenvironment [61]. Some studies suggested that the stability of the mesenchymal phenotype depends on the duration of EC stimulation by various *stimuli* (TGF-*β*1, IL-1*β*, and TNF-*α*) [62, 63]. In human intestinal microvascular endothelial cells (HIMEC) treated simultaneously by TGF-*β*, TNF-*α*, and IL-1*β*, EndoMT appeared completed at 6 days and was prolonged up to 10 days, suggesting then the creation of a stable mesenchymal phenotype [64]. The nature of the extracellular matrix also appears as a determining parameter in the induction of EndoMT, as *in vitro* experiments have demonstrated a significant increase in mesenchymal *α*-SMA expression in ECs cultured on an enriched-fibronectin matrix, but not on the collagen- or gelatin-matrix [43, 65]. These data suggest that the nature of the microenvironment matrix influences the induction of EndoMT and could play a key role in the development of diseases such as fibrosis or cancer [66].

The notion of reversibility/irreversibility has been suggested and evidenced for the epithelial-to-mesenchymal transition (EMT). EMT is reversed by Twist gene silencing [67]. Furthermore, hypoxia-induced EMT in MDA-MB-231 breast cancer cells may be reversed not only by reoxygenation, providing a model for changes that may occur *in vivo* when cancer cells intravasate into the bloodstream or metastasize to the lungs, but also by silencing *µ*PA expression that decreased expression of Vimentin and Snail [68]. Similarly, in Madin–Darby canine epithelial cells, it has been found that Snail overexpression induced EMT, while Snail silencing upregulated epithelial markers and downregulated mesenchymal markers confirming the reversibility of EMT [69]. Taken together, these findings suggest that EndoMT could also be reversible, especially since the mediators mobilized during one and the other of the processes are identical.

Thus, the stability and the reversibility of the mesenchymal phenotype, issued from EndoMT, have been studied *in vitro* by analyzing the evolution of the protein signature after several days of culture in the presence of various *stimuli* (TGF-*β*1, IL-1*β*, and TNF-*α*) [62, 63, 70].

Furthermore, different *in vitro* studies showed that EndoMT is initiated from the first six hours after stimulation and that this process could be reversible for culture times less than 10 days in the presence of proinflammatory cytokines as TGF-*β*1. Furthermore, when EC are treated with cytokines for a period of 20 days, the acquisition of the mesenchymal phenotype is stable over time and irreversible [44, 64].

While partial EMT is well described in the literature, partial EndoMT is, to date, little studied although it constitutes an emerging concept in the field of oncology. Welch-Reardon et al. were the first to suggest this concept by comparing angiogenesis to EndoMT and then by identifying several similarities [71]. Among these, the tip cells that lead emerging sprouts lack apical-basal polarity, degrade the extracellular matrix, and, by definition, are migratory. Moreover, angiogenic ECs do not usually separate from their neighbors and express significantly Slug, a Snail family of zinc-finger transcription factor [71], suggesting that angiogenesis may involve a partial EndoMT [46]. These data have been confirmed in an adenomyosis model in which it has been found that its development is associated with a significant angiogenesis induced by estrogen and dependent on the activation of the Slug-VEGF axis [72]. Finally, more recently, it has been reported, in a model of ovarian carcinoma, that the inhibition of Slug expression decreased significantly the growth of tumor and microvessel density [73]. Taken together, these findings suggest that the same mediators or the same signaling pathways that induce EMT or angiogenesis may also drive ECs toward a mesenchymal phenotype that could be also associated with metastasis. Angiogenesis could represent a partial EndoMT, and we therefore believe that anti-angiogenic drugs may have a dual benefit for treating metastatic cancers, as they could delay metastatic development by inhibiting both angiogenesis and EndoMT.

In summary, EndoMT is characterized by a permanent alteration of the endothelial phenotype evolving toward a mesenchymal phenotype. Specificity of the mesenchymal markers acquired during this transition appears to be dependent not only on the nature of the inducing agents present in the microenvironment but also on the tissue origin of the EC. The reversion of this process may also occur during a short time after its initiation [74], but the acquisition of the mesenchymal phenotype appears stable in a context of chronic induction, as it might be the case in cancer for example.

## 4. Main Signaling Pathways Involved in EndoMT

The TGF-*β* family of proteins comprises several pleiotropic growth factors that play crucial roles in numerous physiological processes including embryogenesis, cellular development and differentiation, immunologic system development, inflammatory response functions, and wound repair. The TGF-*β* superfamily consists of four major subfamilies: the TGF-*β* subfamily, the bone morphogenetic proteins (BMP), the activin and inhibin subfamilies, and a group encompassing various divergent members [75]. TGF-*β*1, -*β*2, and -*β*3 are three distinct isoforms, which have been extensively found in mammal tissues. TGF-*β* signals through TGF-*β* receptors (T*β*Rs) I and II to activate downstream signaling pathways [76]. In the absence of ligand, T*β*RI and T*β*RII exist as monomers, homodimers, or heterodimers on the cell surface. Ligand binding promotes formation of a tetrameric complex between T*β*RII dimers and two T*β*RIs. TGF-*β* binds specifically to the constitutively active T*β*RII, which activates T*β*RI by phosphorylating the glycine/serine-rich domain. Activated T*β*RI then phosphorylates downstream effectors to induce signal transduction [77]. Induction of EndoMT through TGF-*β* seems to involve two distinct signaling pathways: (i) one leading to an increase of Snail-1, one of the main transcription factors that regulate EndoMT together with Slug and Twist, and (ii) one recruiting the Smad pathways [78]. Furthermore, TGF-*β* signals through both canonical Smad-dependent and non-canonical Smad-independent pathways. Considering the canonical pathway, following ligand binding, the type II receptor phosphorylates the type I receptor, which in turn phosphorylates the receptor-regulated Smads (R-Smads) (Smad-2 and -3). When activated, R-Smads associate with Smad-4, a common-partner Smad (Co-Smad), and translocate to the nucleus to control the transcription of the target gene [79]. Smad-7 acts as a negative feedback and regulates Smad signaling by forming a stable complex with type I receptors, therefore leading to inhibition of R-Smad phosphorylation and the heterocomplex formation between R-Smads and Co-Smad [79]. This heterocomplex translocates into the nucleus, where it regulates the transcription of target genes [80], among which are the differentiation transcription factors Snail, Twist, and Slug.

**Figure 2:**
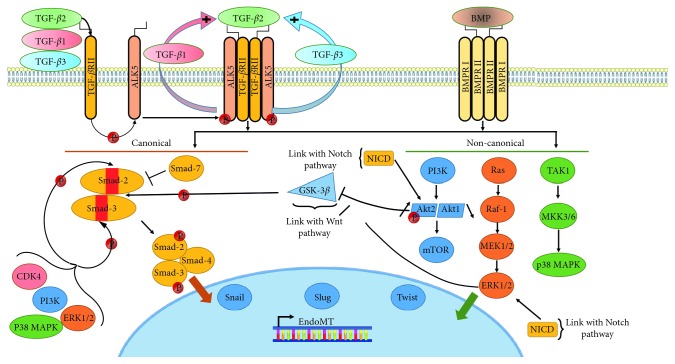
Transforming growth factor-*β-* (TGF-*β-*) induced EndoMT. Upon stimulation by TGF-*β*1, -2, or -3, type-2 TGF-*β* receptors phosphorylate ALK5 (type-1 TGF-B receptor) and associate into a heterotetrameric structure which induce Smad-2/3/4 complex formation and translocation to the nucleus. Stimulation by TGF-*β*1 or -3 induces a paracrine loop toward a TGF-*β*2 stimulation. Smad-7 acts as an inhibitor of Smad association and serves as a negative retro control. TGF-*β* signaling also induces phosphorylation of ERK 1/2 (extracellular signal-regulated kinases 1/2) and p38 MAPK (p38 mitogen-activated protein kinase). The BMP receptors can also trigger upon stimulation by the BMP ligand canonical Smad pathway and non-canonical ERK pathway. Inside the nucleus, all actors involved stimulate the activity of the transcription factor, mainly Snail, Slug, and Twist, thus initiating EndoMT by promoting transcription of mesenchymal markers and diminishing transcription of endothelial markers. TGF-*β* signaling crosstalk with several others pathways, including Notch which promotes ERK 1/2 activity and Akt2 isoform activity which will then inhibit GSK-3*β*. GSK-3*β*, ERK, PI3K, P38, and also CDK4 can phosphorylate Smad-2 and -3 on specific residues in its linker region (in red) promoting Smad signaling in a canonical-independent manner. However, it is worth to note that some studies report the inhibiting effect of linker region phosphorylation, and that the specific effects of this phosphorylation site seem to be cell-type dependent.

In addition to this canonical pathway, TGF-*β* isoforms are also able to activate Smad independent or non-canonical pathways, including mitogen-activated protein kinases and phosphoinositide 3-kinase/AKT (PI3K/AKT) signaling pathways [81]. Indeed, TGF-*β* signaling also activates numerous serine/threonine kinases that phosphorylate Smad-2 in its linker region. Interestingly, in primary-cultured bovine aortic ECs, it has been reported that TGF-*β*-mediated phosphorylation of individual serine/threonine sites in the linker region of Smad-2 occurs in a highly specific manner by kinases [82] (Figure 2).

TGF-*β* is considered the most important regulator of both EMT in cancer [83] and EndoMT in cardiovascular development [84] or disease as well as in cancer [60, 84]. However, it appears that the three isoforms of TGF-*β* did not have the same efficacy to induce EndoMT, according to the tissue origin of the EC and to the pathophysiological context (heart development, fibrosis, and cancer). TGF-*β*1 has been firstly reported to be the main and unique regulator of EndoMT in a mouse atriovascular canal (AVC) model [85], while TGF-*β*2 and TGF-*β*3 were shown to cooperate to mediate EndoMT in cultured chick AVC explant cells. In this study, it has been also reported that TGF-*β*2 mediates initial endothelial cell-cell separation, while TGF-*β*3 is required for the cell morphological change that enables the migration of cells into the underlying extracellular matrix [21]. Of the different isoforms of TGF-*β*, TGF-*β*2 appeared to be the one that induced EndoMT. Thus, in different EC models including the mouse pancreatic microvascular endothelial cells (MS-1), it has been noted that activation of Smad signals by TGF-*β*2 has dual effects on the activation of Rho signals and myocardin-related transcription factor-A (MRTF-A), leading to the mesenchymal transition of MS-1 endothelial cells [86].

Recent studies have investigated the dose effect of TGF-*β*1, TGF-*β*2, and TGF-*β*3 on the induction of EndoMT in invasive colon cancer and have shown that TGF-*β*2, by increasing the expression of mesenchymal markers N-cadherin and *α*-SMA, is the most potent inducer of EndoMT in this model [87]. These findings have been confirmed in endothelial cells HMEC-1 grown in conditioned media from invasive colon cells. An increased production of TGF-*β*1 correlates with a significant EndoMT and is associated with an increase of *β*3-tubulin expression and phosphorylation [87]. Furthermore, it has also been highlighted that TGF-*β*1- and TGF-*β*3-induced EndoMT requires a paracrine loop involving TGF-*β*2 [88].

Concerning the non-canonical pathway or Smad-independent pathway, TGF-*β*2 has been shown to activate PI3K/Akt/mTOR, ERK 1/2, and P38 MAPK pathways. These signaling pathways appear then necessary to promote the increased expression of transcription factor Snail [78]. This direct regulation of Snail by TGF-*β*2 has been highlighted in a study conducted on mouse embryonic stem cell-derived endothelial cells (MESECs), where TGF-*β*2-induced EndoMT, characterized by a decrease in the expression of the endothelial marker claudin 5 and an increase in expression of the mesenchymal *α*-SMA [89].

Snail is described as one of the major transcription factors involved in cell plasticity, suppressing cell adhesion and promoting EMT [90, 91]. Interestingly, studies have shown that overexpression of Snail in cells is sufficient to induce EMT. If Snail alone is able to induce EMT, additional mechanisms are involved to mediate change in endothelial morphology in the case of EndoMT. One of them relies on the inhibition of GSK-3*β*. This protein was identified as a regulator of Snail activity in TGF-*β*2-induced-EndoMT [78]. Snail protein stability and nuclear translocation are inhibited through phosphorylation by GSK-3*β* [92]. In human cardiac endothelial cells (HCMECs), it has been shown that inhibition of GSK-3*β* by TGF-*β*2-induced PI3K signaling allows Snail to induce EndoMT by transcriptional modulation. PI3K is also necessary for controlling Snail gene expression, demonstrating a dual role for this pathway in mediating EndoMT [78].

Hypoxia has also been described as a potent inducer of EndoMT. HIF-1, the main effector of the hypoxia pathway, is responsible for driving the expression of VEGF-A to promote angiogenesis. Previous research has identified an active hypoxia response element (HRE) within the VEGF promoter and implicated that TGF-*β* can cooperate with hypoxia to enhance VEGF transcription [93], providing some of the first evidence that these two factors can work together to drive angiogenesis. In addition, several studies suggest that hypoxia regulates the expression of TGF-*β*1, -2, and -3 [94–96]. Finally, hypoxia also induces the expression of EndoMT-associated transcription factor Snail and Slug [97]. Both of these factors are induced by TGF-*β* in ECs [98] and were demonstrated to drive EndoMT associated with the sprouting phase of angiogenesis [71].

The Wnt/*β*-catenin canonical pathway is another *stimulus* for inducing EndoMT [33, 99]. Wnt signaling is a complex collection of signal transduction pathways mediated by multiple signaling molecules and is critically important for developmental processes, including cell proliferation, differentiation, and tissue patterning [100].

**Figure 3:**
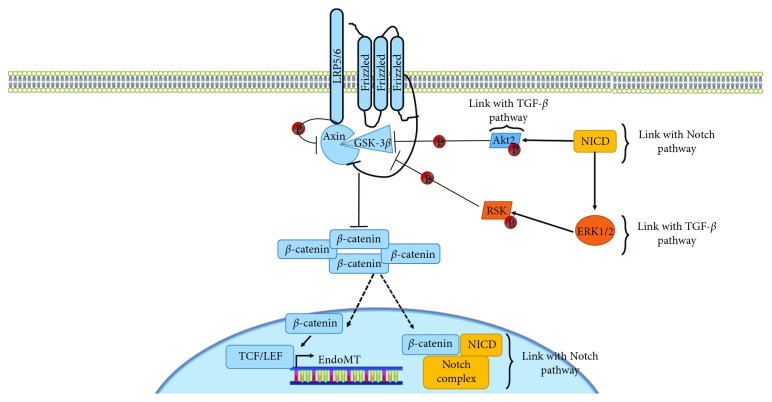
Wnt induction of EndoMT. Wnt bound the extracellular part of the Frizzled receptor, while LRP5/6 serves as co-receptors. This causes the complex Axin-GSK-3*β* to bind to the cytoplasmic tail of LRP5/6. Therefore, this complex is no longer able to assure the degradation of *β*-catenin, which accumulates in the cytoplasm and translocates into the nucleus to stimulate the activity of transcription factors of the TCF/LEF families. The Wnt pathway crosstalk with several other pathways: Akt 2 can phosphorylate and thus inhibit GSK-3*β*. Moreover, RSK phosphorylation ERK 1/2-dependent RSK phosphorylation leads to inhibition of GSK-3*β*.

Wnt signaling activates cytoplasmic effectors and regulates the transcription of target genes (Figure 3). *β*-Catenin is a downstream effector of the Wnt signaling that accumulates in the cytoplasm and eventually translocates into the nucleus to act as a transcriptional coactivator for TCF/LEF transcription factors family members [101]. While the detailed molecular events and signaling pathways initiating EndoMT have not been clearly elucidated, this canonical *β*-catenin-dependent Wnt pathway has been found to be involved in EndoMT in both myocardial infarction [99, 102] and in oral squamous cell carcinoma [103].

Reactive oxygen species (ROS) have emerged as an important factor affecting several cancer hallmarks [104]. ROS are involved in the acquisition of self-sufficiency in proliferation signals and in the development of a more aggressive phenotype through matrix metalloproteinase (MMP) secretion and regulation of cellular plasticity. The ROS family includes several molecules, such as hydroxyl radical (^·^OH), superoxide radical (O_2_^−^), hydrogen peroxide (H_2_O_2_), and peroxynitrite (OONO^−^), which are produced by normal and pathogenic oxygen metabolism. Several studies have evaluated the role of oxidative stress in the control of EndoMT in endothelial dysfunction [105], atherosclerosis [70], or renal failure secondary to renal ischemia [106]. Treatment with 0.1 to 10 *μ*M H_2_O_2_ triggered the transformation process in primary EC, as observed by changes in endothelial and mesenchymal markers expression. This effect is mediated by TGF-*β*1 secretion and is dependent on Smad-3 activation [105]. Furthermore, it has been reported in HUVEC treated by TGF-*β*1 that inhibition of oxidative stress by kallistatin, a plasma protein distributed in blood vessels, is correlated with a significant decrease of EndoMT [35]. In the context of massive oxidative stress such as tissue exposure to ionizing radiation, it has been established that irradiated intestinal endothelial cells undergo endothelial-to-mesenchymal transition. Therefore, radiation-induced EndoMT participates in radiotherapy-induced gut damage such as proctitis [49]. While these findings confirmed a main role of ROS to induce EndoMT in ECs, little is known about the actual impact of oxidative stress in triggering EndoMT in cancer. However, cancer-associated fibroblasts (CAFs) issued from EndoMT could be important intermediaries through their capacity to significantly produce ROS [107]. Indeed, because ROS are hallmarks of inflammation, known as a common state in tumoral stroma, we can hypothesize a strong link between ROS and EndoMT in the context of cancer.

**Figure 4:**
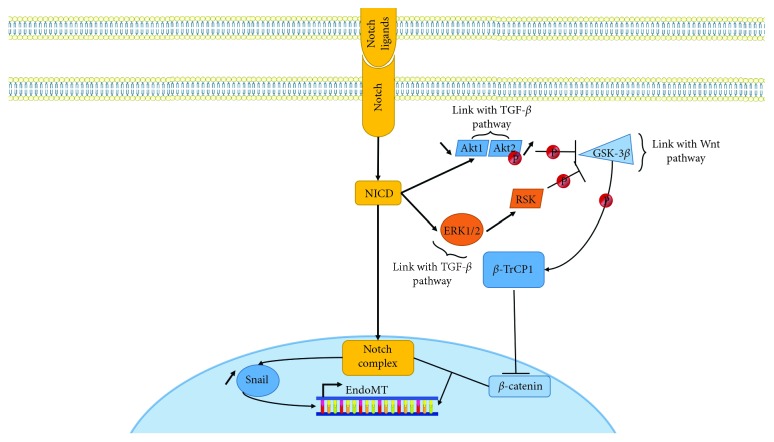
Notch induction of EndoMT. Notch receptors' family interacts with diverse ligands via a cell-to-cell contact mechanism. The signal is mediated by the Notch intracellular domain (NICD) through the nucleus to activate a complex of inducers (RBPJ/CBF1/Su(H)) which in turn activates the transcription of genes implicated in EndoMT. This complex also stabilizes the Snail protein. The Notch pathway crosstalks with several other pathways: NICD increases the Akt 2 expression which inhibits GSK-3*β*. NICD also activates ERK 1/2, which activates RSK leading to GSK-3*β* downregulation. The nuclear Notch complex also interacts with *β*-catenin, increasing its transcription activity.

Taken together, the studies presented in this review clearly illustrate the participation of different signaling pathways in the modulation of EndoMT. However, it is not excluded that other mediators could also be involved. Among these, Notch was identified in EndoMT observed in the cardiovascular pathophysiological context. Notch is defined as a transmembrane receptor and a transcription factor [108]. The Notch signaling is an evolutionary conserved pathway that plays an essential role in both invertebrates and vertebrates, by controlling cellular fate, cell growth, and differentiation. Notch signaling is pleiotropic, influencing embryogenesis, differentiation, and homeostasis in adult tissue, and it contributes to the plasticity and functionality of different cell types. Perturbations in the Notch signaling pathway have been associated with various genetic disorders and cancers [109]. The role of Notch in the control of EndoMT has been mainly shown during heart valves development, arterial-venous differentiation, and remodeling of the primitive vascular plexus [29]. In the embryonic heart, Notch has been identified to promote a TGF-*β*-mediated EndoMT that leads to development of cardiac valvular. This process is explained by an induction of Snail-1 expression and activity and a downregulation of VE cadherin expression [110]. This TGF-*β*/Notch link has been confirmed in an aortic EC model as active Notch expression promotes EndoMT, resulting in downregulation of VE cadherin and upregulation of mesenchymal genes such as those for fibronectin and Snail-1/2. Furthermore, TGF-*β*1 was reported to exacerbate Notch effects by increasing Snail-1 and fibronectin activation [111] (Figure 4). Furthermore, the endothelial overexpression of the transcription factor Hey-2, a well-known Notch effector, has been shown to induce EndoMT in a preclinical model of radiation-induced proctitis [112].

Few studies have assessed the role of Notch in EndoMT during tumorigenesis. Thus, from a xenograft tumor assay of two breast tumor cell lines MDA-MB231 or MCF-7 in NOD/SCID mice, an education of EC by tumors cells through a crosstalk between Notch and TGF-*β* pathways has been reported. This cooperation generated the formation of a transient mesenchymal/endothelial niche, associated with a significant increase in tumor proliferation, stemness, and invasiveness [113].

## 5. EndoMT: A Pathophysiological Process Promoting Tumorigenesis

The process of EndoMT initially described in physiological cardiac development has also been identified in the pathological context of cardiac and pulmonary fibrosis [11], atherosclerosis [12], and vascular calcification [114]. Furthermore, increasing evidence implies the process of EndoMT in the context of cancer as a relevant contributor of the tumor microenvironment plasticity.

### 5.1. EndoMT and Cancer-Associated Fibroblasts (CAFs)

It is clearly established that ECs through the EndoMT process is an important source of CAFs. *In vivo*, use of the gold standard strategy to explore cell lineage conversion, namely, the Cre-LoxP genetic lineage labeling system technology [52], allowed to prove that up to 40% of CAF in pancreas cancer or melanoma model [17] results from EndoMT [46]. These cells are known to facilitate cancer progression [17, 37]. CAFs are now identified as the major contributor to tumor growth and metastatic dissemination [77], mainly through their secretome and release of classical growth factors and chemokines shown to influence different aspects of tumor cell behavior [115]. CAFs issued from EndoMT acquire their activated state and maintain it by various mechanisms including genetic or epigenetic mutations or under the persistent effect of growth factors or specific cytokines produced by tumor microenvironment [79].

During primary cancer progression, CAFs communicate with cancer cells through the secretion of growth factors, chemokines, and cytokines. For instance, CAF-derived TGF-*β*, epidermal growth factor (EGF), fibroblast growth factor (FGF), vascular endothelial growth factor (VEGF), and matrix metalloproteinase (MMP) have been implicated in epithelial cancer progression [116, 117]. Furthermore, CAFs are able to provide potential oncogenic signals: (i) CAF-derived TGF-*β* participates in acceleration of cancer cell invasion, and (ii) CAF-derived growth factors and angiogenic factor VEGF can stimulate cancer progression, including angiogenesis [118]. More recently, the role of CAF in tumor angiogenesis has been described in a C8161-HA mouse melanoma model. In this study, the authors established a link between tumor development and inhibition of SERPIN F1 production by activated fibroblasts from tumor microenvironment. This latest inhibition was associated with an increase of angiogenesis and a strong expression of proangiogenic factors [119].

### 5.2. EndoMT and Extravasation of Tumor Cells

Evidence suggests that ECs are not passive actors during transendothelial migration of cancer cells, as this passage requires profound changes in endothelial junctional protein expression, signaling, permeability, and contractility. EndoMT leads to a deep reorganization of microvessels with a cytoskeletal remodeling, an increase in endothelial barrier permeability linked to loss of adhesion molecules (claudins and VE-cadherin). This active process in the endothelial compartment is compatible with the extravasation of cancer cells, which is the first step of the metastatic process [120]. This hypothesis has been confirmed in a study which evaluated the role of EndoMT induced by TGF-*β*1 on various ECs (brain endothelial cells and HUVEC) during melanoma metastatic extravasation. Stimulation of ECs with activated cancer cell line-conditioned medium resulted in TGF-*β*-dependent decrease of transendothelial electrical resistance (TEER), increase in adhesion between metastatic and ECs, and enhanced transendothelial migration of melanoma cells. These findings suggested that EndoMT may be necessary for an optimal metastatic transendothelial migration and may be one of the potential mechanisms occurring during the complex phenomenon of metastatic extravasation [121].

## 6. EndoMT and Response to Therapies

As described in this review, EndoMT is found in tumors and is mainly induced by factors issued from the tumor itself or from its educated microenvironment, such as TGF-*β*. This phenotypic transition is a unique source of CAFs [17] and may also be part of a facilitated extravasation of cancer cells into the blood circulation [121]. Cancer therapies may also be regulating factors of EndoMT. Histone-deacetylase HDAC inhibitor valproic acid, currently under clinical investigation for anticancer therapy, has recently been shown to induce EndoMT *via* a TGF-*β*1 signaling pathway [15]. On the contrary, a conjugate of temozolomide and perillyl alcohol, used in a glioblastoma model, inhibits EndoMT and reverts the mesenchymal phenotype of tumor-associated brain EC [122]. Exposure to ionizing radiation also leads to a phenotypic conversion of EC in the colon and lung carcinoma preclinical models [57] but also in normal rectal tissues, leading then to radiation-induced fibrosis [49]. Increasing evidence highlights the importance of EndoMT in tumor progression, favoring metastasis and being an important source of CAFs. Furthermore, the role of EndoMT in cancer resistance to therapies appears as a novel emerging field with scarce but exciting studies. As mentioned above, radiation induces EndoMT that awakes dormant cancer stem cells from hypoxic regions and polarizes tumor-associated macrophages TAM toward an M2 phenotype, therefore conferring tumor radio-resistance and promoting tumor progression [57]. Resistance to chemotherapies cisplatin and gefitinib in a multicellular lung tumor spheroid model is alleviated when EndoMT in ECs in the spheroid is reversed, implying EndoMT as a resistance factor [123]. In an invasive colon cancer model, EndoMT culminates in the generation of CAF-overexpressing tubulin-3, a known factor of resistance to taxanes-type of chemotherapeutics [87]. The resulting cells of the EndoMT process, namely, the CAF, are well described for their roles in cancer resistance, recently reviewed [16]: they produce soluble factors (IL-6 and IL-8) associated with chemiresistance, and they can control chemotherapy uptake either by reducing expression of drug transporters or by trapping active drugs, limiting their availability to the tumor. CAFs are also known to limit oxidative stress-induced by chemotherapy and therefore to protect the tumor cell from ROS-induced apoptosis. Furthermore, in breast and lung cancers, CAFs have been shown to sustain cancer stemness, by promoting a survival niche for cancer stem cells [124]. Several studies reinforce this notion of a link between EndoMT and stemness. Indeed, radiation-induced EndoMT has been shown to reactivate dormant cancer stem cells CD44v6^+^, driving then tumor regrowth [57]. In a non-tumoral model of pulmonary arterial hypertension (PHA), that share similarities with carcinogenesis (excessive proliferation, apoptotic resistance, and inflammation), expression of the stemness marker CD44v is induced in pulmonary EC undergoing EndoMT and is associated with the increased level of antioxidant GSH molecules [125]. Analogy between PAH and carcinoma suggests that EndoMT could favor survival of either mesenchymal cells (PHA) or tumor cells (carcinoma) in condition of important oxidative stress such as that induced by chemotherapy and radiotherapy. Several works pointed that EndoMT could also induce an abnormal recruitment of pericytes [57] and could give rise to pericyte-like cells within the tumor and abnormally cover the vasculature [58]. These populations of pericytes have been proposed as a signature of tumors refractory to anti-VEGF therapy in at least two different cancer models (pancreas [126] and melanoma [127]), highlighting an implication of EndoMT in tumor stromal resistance. The role of EndoMT needs to be deeply explored and fully understood as this process of cellular plasticity could be envisaged in a close future as the therapeutic strategy.

## 7. Conclusion

Taken together, the studies presented in this review clearly identify plasticity of ECs as a pillar of tumor development through modification of their phenotype. This plasticity is involved in tumorigenesis and metastatic progression and appears relevant in resistance therapy. Evidence suggests the existence of a complex signaling network involving TGF-*β*, Wnt/*β*-catenin, and Notch pathways that mediate and control EndoMT. The complexity of these pathways and their potential interconnections suggest that further studies are necessary to better understand their roles in EndoMT in both animal tumor models and in human cancer. Thus, future efforts should be devoted to the exploration of molecular mechanisms involved in this process. These efforts would eventually lead to the development of novel therapeutic approaches, targeting this microenvironmental plasticity to improve tumor treatment and limit metastatic dissemination and resistance to various anti-tumor therapies.

## Figures and Tables

**Table 1:** Comparison of main features during EMT and EndoMT.

	EMT	EndoMT
Cells	Epithelial cells	Endothelial cells
Induction mediators	TGF-*β* [23], Wnt/*β*-catenin [24], Notch [25]	TGF-*β* [26], Wnt/*β*-catenin [27], hypoxia [13], oxidative stress [28], Notch [29]
Epithelial/endothelial markers	E-cadherin, N-cadherin [30, 31]	VE cadherin, CD31/PECAM-1, vWF
Mesenchymal markers	*α*-SMA, FSP-1, vimentin	
Transcription factors	Slug, Snail-1 and -2, Twist	
